# Sequential Treatment with Regorafenib and Trifluridine/Tipiracil in Refractory Metastatic Colorectal Cancer

**DOI:** 10.3390/life16040564

**Published:** 2026-03-30

**Authors:** Min-Chi Cheng, Po-Huang Chen, Yu-Guang Chen, Shiue-Wei Lai, Jia-Hong Chen, Ming-Shen Dai, Ping-Ying Chang

**Affiliations:** 1 Division of Hematology and Oncology, Department of Internal Medicine, Tri-Service General Hospital, National Defense Medical University, Taipei 114, Taiwan; chengminchi123@gmail.com (M.-C.C.);; 2 Department of Oncology, Tri-Service General Hospital, National Defense Medical University, Taipei 114, Taiwan

**Keywords:** metastatic colorectal cancer, regorafenib, trifluridine/tipiracil, treatment sequencing, propensity score, inverse probability of treatment weighting

## Abstract

Background: The optimal sequencing of regorafenib and trifluridine/tipiracil (FTD/TPI) in refractory metastatic colorectal cancer (mCRC) remains uncertain, particularly in Asian populations. Methods: We retrospectively analyzed 110 patients with mCRC who sequentially received both agents between 2011 and 2025. Patients were categorized into regorafenib followed by FTD/TPI (Rego → FTD/TPI, *n* = 88) and FTD/TPI followed by regorafenib (FTD/TPI → Rego, *n* = 22). Co-primary endpoints were time to treatment discontinuation (TTD) and overall survival (OS). Propensity score-based weighting methods, including stabilized inverse probability of treatment weighting (primary analysis), were used to adjust for baseline imbalances. Multivariable Cox regression was performed as a sensitivity analysis. Results: No statistically significant differences were observed between treatment sequences. In the primary analysis, the hazard ratio (HR) for TTD was 1.01 (95% CI 0.71–1.43), and for OS was 1.19 (95% CI 0.67–2.12), with FTD/TPI → Rego as reference. Median TTD was 6.8 versus 8.9 months, and median OS was 14.6 versus 20.2 months, respectively. Conclusions: Clinical outcomes were comparable regardless of treatment order, supporting individualized sequencing decisions in refractory mCRC.

## 1. Introduction

Colorectal cancer (CRC) remains the third most prevalent cancer globally and the second leading cause of cancer-related mortality, with more than 1.9 million new cases diagnosed annually worldwide [1,2]. Despite significant advances in screening, early detection, and multimodal treatment strategies, approximately 25% of patients present with synchronous metastatic disease at initial diagnosis, and more than half eventually develop metastases during their disease course [3,4]. For patients with unresectable metastatic colorectal cancer (mCRC), the therapeutic landscape has evolved considerably over the past two decades, with first- and second-line treatments typically consisting of fluoropyrimidine-based chemotherapy doublets or triplets combined with targeted agents including anti-vascular endothelial growth factor (VEGF) and anti-epidermal growth factor receptor (EGFR) therapies [5,6].

Following progression on standard first- and second-line therapies, a substantial proportion of patients maintain adequate performance status and remain candidates for further systemic treatment [7]. In this refractory setting, two oral agents have emerged as established third-line options: regorafenib, a multikinase inhibitor, and trifluridine/tipiracil (FTD/TPI, also known as TAS-102), a novel oral fluoropyrimidine-based nucleoside analog [8,9]. Both agents have demonstrated survival benefits compared with best supportive care in pivotal randomized controlled trials and have been incorporated into international guidelines for the management of chemorefractory mCRC [10,11].

Regorafenib is an oral multikinase inhibitor that targets multiple signaling pathways implicated in tumor angiogenesis (VEGFR1-3, TIE2), oncogenesis (KIT, RET, RAF1, BRAF), and the tumor microenvironment (PDGFR, FGFR) [12]. The phase III CORRECT trial established regorafenib as the first small-molecule agent to demonstrate a survival advantage in treatment-refractory mCRC, with median overall survival (OS) of 6.4 months versus 5.0 months for placebo (HR 0.77; 95% CI 0.64–0.94; *p* = 0.0052) [13]. These findings were subsequently validated in the CONCUR trial, which specifically evaluated regorafenib in Asian patients and demonstrated an even more pronounced survival benefit (median OS 8.4 vs. 6.3 months; HR 0.55; 95% CI 0.40–0.77; *p* < 0.001) [14].

FTD/TPI is a novel oral combination comprising trifluridine, a thymidine-based nucleoside analog that is incorporated directly into DNA causing strand breaks, and tipiracil hydrochloride, a thymidine phosphorylase inhibitor that prevents the degradation of trifluridine and maintains its systemic bioavailability [15]. The mechanism of action differs from conventional fluoropyrimidines such as 5-fluorouracil and capecitabine, which primarily act through RNA incorporation. The phase III RECOURSE trial randomized 800 patients with refractory mCRC to FTD/TPI or placebo and demonstrated significant improvements in median OS (7.1 vs. 5.3 months; HR 0.68; 95% CI 0.58–0.81; *p* < 0.001) and progression-free survival (2.0 vs. 1.7 months; HR 0.48; 95% CI 0.41–0.57; *p* < 0.001) [16]. The survival benefit was maintained regardless of KRAS mutation status and in patients who had previously received regorafenib [16,17].

Despite the availability of both agents for late-line treatment, there remains no consensus on the optimal sequencing strategy. Network meta-analyses comparing regorafenib and FTD/TPI have consistently suggested similar efficacy between the two agents in terms of OS and progression-free survival [18,19]. However, their distinct toxicity profiles—with regorafenib primarily causing hand-foot skin reaction, fatigue, and hypertension, while FTD/TPI predominantly induces myelosuppression, particularly neutropenia—provide clinical rationale for considering treatment sequence based on individual patient factors [20,21]. The practice-changing results of the SUNLIGHT trial established FTD/TPI plus bevacizumab as a new standard of care for third-line treatment (median OS 10.8 vs. 7.5 months; HR 0.61; 95% CI 0.49–0.77; *p* < 0.001) [22,23], further emphasizing the importance of understanding optimal treatment sequencing strategies in this setting.

Recent evidence from the randomized phase II SOREGATT/PRODIGE 68 trial provided the first prospective data comparing treatment sequences in patients with refractory mCRC, finding no significant difference in outcomes between the two sequences [24]. However, the generalizability of these findings to Asian populations with potentially different pharmacokinetic profiles, treatment tolerability, and healthcare delivery systems requires validation. Therefore, we conducted this retrospective cohort study at Tri-Service General Hospital to compare the clinical outcomes of two sequential treatment strategies in Taiwanese patients with mCRC.

## 2. Materials and Methods

### 2.1. Study Design and Population

This retrospective cohort study was conducted using the cancer registry database of Tri-Service General Hospital (TSGH), a tertiary medical center in Taipei, Taiwan [25]. We identified all patients with histologically confirmed mCRC who received both regorafenib and FTD/TPI between January 2011 and December 2025. Patients were included if they had received both agents sequentially (i.e., one agent followed by the other upon progression or intolerance) and had complete baseline and outcome data. Patients were excluded if they received both agents concurrently, had incomplete treatment records, or were lost to follow-up before experiencing the primary outcome events.

### 2.2. Treatment Groups

Patients were classified into two groups based on the sequence of treatment administration: (1) the Rego → FTD/TPI group, comprising patients who received regorafenib as the initial third-line agent followed by FTD/TPI upon progression or intolerance; and (2) the FTD/TPI → Rego group, comprising patients who received FTD/TPI first followed by regorafenib. The choice of initial treatment was at the discretion of the treating physician and was not randomized.

### 2.3. Outcomes

The co-primary outcomes were time to treatment discontinuation (TTD) and overall survival (OS). TTD was defined as the time from initiation of the first third-line agent (either regorafenib or FTD/TPI) to discontinuation of the second agent for any reason, including disease progression, intolerable toxicity, patient decision, or death. OS was defined as the time from initiation of the first third-line agent to death from any cause. Patients who were alive at the last follow-up were censored at that time point. Adverse events were assessed as a secondary outcome. Clinical Grade 3 or higher adverse events occurring during the second-agent treatment period were ascertained through retrospective chart review and graded according to the Common Terminology Criteria for Adverse Events (CTCAE) version 5.0. Hematologic toxicities, including neutropenia, anemia, and thrombocytopenia, were additionally graded from available complete blood count (CBC) laboratory data obtained during the treatment period. Differences in adverse event rates between treatment sequences were compared using Fisher’s exact test.

### 2.4. Covariates

Baseline characteristics assessed at the time of initiating the first third-line agent included: demographic variables (age, sex), Eastern Cooperative Oncology Group performance status (ECOG PS), Charlson Comorbidity Index (CCI), tumor characteristics (primary tumor location classified as left-sided, right-sided, or rectum; KRAS mutation status), metastatic sites (liver, lung, and bone), prior treatment history (number of prior lines of therapy, prior anti-EGFR therapy exposure), and prior colectomy status. Additionally, bevacizumab combination with FTD/TPI was included as a covariate given its established efficacy in the SUNLIGHT trial and its potential to influence treatment outcomes.

### 2.5. Statistical Analysis

Baseline characteristics were summarized using means and standard deviations for continuous variables and frequencies and percentages for categorical variables. Standardized mean differences (SMDs) were calculated to assess balance between treatment groups, with |SMD| < 0.1 indicating adequate balance [26].

To address confounding by indication in this observational study, we employed propensity score-based methods [27]. The propensity score, defined as the probability of receiving the Rego → FTD/TPI sequence conditional on baseline covariates, was estimated using a logistic regression model that included all baseline covariates listed above plus bevacizumab combination status [28]. The inclusion of bevacizumab as a covariate was based on its clinical importance following the SUNLIGHT trial results and its potential to influence treatment sequencing decisions. We then applied three weighting approaches: (1) inverse probability of treatment weighting (IPTW), where each patient is weighted by the inverse of the probability of receiving their actual treatment [29]; (2) stabilized IPTW (S-IPTW), which multiplies the IPTW weights by the marginal probability of treatment to reduce variance inflation [30]; and (3) overlap weights, which assign weights proportional to the probability of receiving the alternative treatment, thereby emphasizing patients in the region of clinical equipoise and mitigating the influence of extreme propensity scores [31,32].

For IPTW and S-IPTW analyses, weights were truncated at the 1st and 99th percentiles to limit the influence of extreme weights. Covariate balance after weighting was assessed using SMDs. Treatment effects were estimated using weighted Cox proportional hazards regression models with robust (sandwich) standard errors [33]. Hazard ratios (HRs) and 95% confidence intervals (CIs) were reported, with the FTD/TPI → Rego group as the reference. In addition to propensity score-weighted analyses, we performed multivariable Cox regression adjusting for all baseline covariates as a sensitivity analysis.

Exploratory subgroup analyses were performed to examine whether treatment effects differed across clinically relevant subgroups defined by age (<65 vs. ≥65 years), sex, ECOG PS (0 vs. ≥1), primary tumor location (left-sided vs. right-sided), KRAS mutation status, presence of liver metastasis, and bevacizumab combination use. To assess the robustness of our findings to potential unmeasured confounding, we calculated E-values for the primary outcomes [34]. All statistical analyses were performed using R version 4.3.0. Two-sided *p*-values < 0.05 were considered statistically significant.

## 3. Results

### 3.1. Patient Characteristics

**Figure 1:**
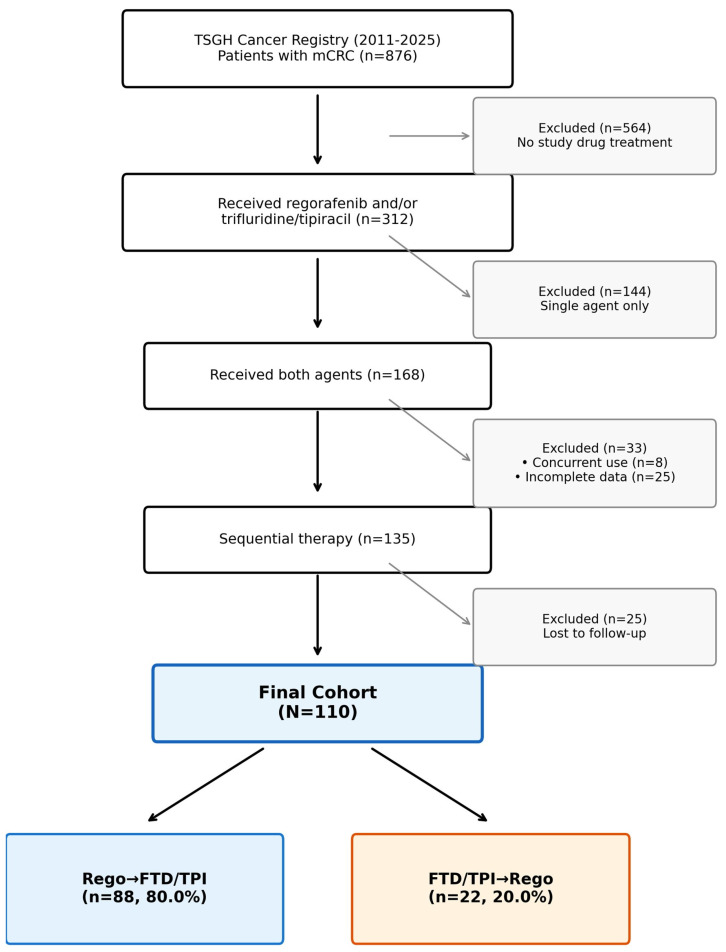
Study flowchart. From the Tri-Service General Hospital cancer registry (20112025), 876 patients with metastatic colorectal cancer (mCRC) were screened. After applying inclusion and exclusion criteria, 110 patients who received both regorafenib and trifluridine/tipiracil (FTD/TPI) sequentially were included in the final analysis. Patients were classified into two groups based on treatment sequence: Regorafenib → FTD/TPI (*n* = 88) and FTD/TPI → Regorafenib (*n* = 22).

From the TSGH cancer registry, we identified 876 patients with mCRC between 2011 and 2025. Of these, 312 patients received regorafenib and/or FTD/TPI, and 168 patients received both agents. After excluding 33 patients who received concurrent therapy or had incomplete data and 25 patients who were lost to follow-up, 110 patients met the inclusion criteria for the final analysis (Figure 1). Among these, 88 patients (80.0%) received the Rego → FTD/TPI sequence and 22 patients (20.0%) received the FTD/TPI → Rego sequence.

Baseline characteristics of the study population are summarized in Table 1. The mean age was 63.9 ± 10.2 years, and 60.9% were male. Approximately half of the patients (50.9%) had ECOG PS 0, and the mean CCI was 7.8 ± 2.0. Left-sided primary tumors were most common (65.5%), and 41.8% of patients harbored KRAS mutations. Liver metastasis was present in 78.2% of patients. FTD/TPI was combined with bevacizumab in 32.7% of patients overall, with a higher rate in the FTD/TPI → Rego group (45.5% vs. 29.5%). Several baseline imbalances were noted between treatment groups, including age (SMD = −0.28), liver metastasis (SMD = −0.30), and tumor location (SMD = −0.21).

### 3.2. Propensity Score Analysis

The propensity score model, which included 14 covariates (13 baseline characteristics plus bevacizumab combination status), showed adequate discrimination (Supplementary Table S1). The distribution of propensity scores demonstrated substantial overlap between treatment groups (Supplementary Figure S1), with mean propensity scores of 0.824 for the Rego → FTD/TPI group and 0.705 for the FTD/TPI → Rego group. After S-IPTW weighting, balance was substantially improved, with 11 of 14 covariates achieving |SMD| < 0.1 (Supplementary Table S2, Figure S2). The distribution of weights showed that S-IPTW successfully reduced extreme weights compared with conventional IPTW (mean S-IPTW weight 1.00 vs. mean IPTW weight 2.00; Supplementary Table S5, Figure S4).

### 3.3. Primary Outcomes

**Figure 2:**
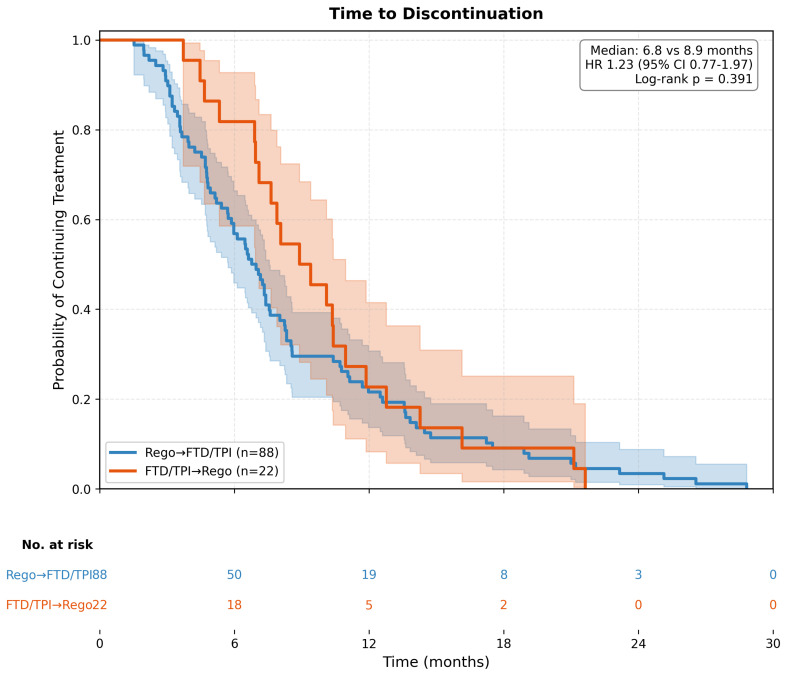
Kaplan–Meier curves for time to discontinuation (TTD) by treatment sequence (unadjusted analysis). The curves compare TTD between patients who received Regorafenib → FTD/TPI (blue line, *n* = 88) versus FTD/TPI → Regorafenib (red line, *n* = 22). Shaded areas represent 95% confidence intervals. Median TTD was 6.8 months (95% CI 5.6–8.3) for Rego → FTD/TPI and 8.9 months (95% CI 6.4–12.5) for FTD/TPI → Rego. The unadjusted hazard ratio was 1.23 (95% CI 0.77–1.97; log-rank *p* = 0.389). Numbers at risk are shown below the x-axis.

**Figure 3:**
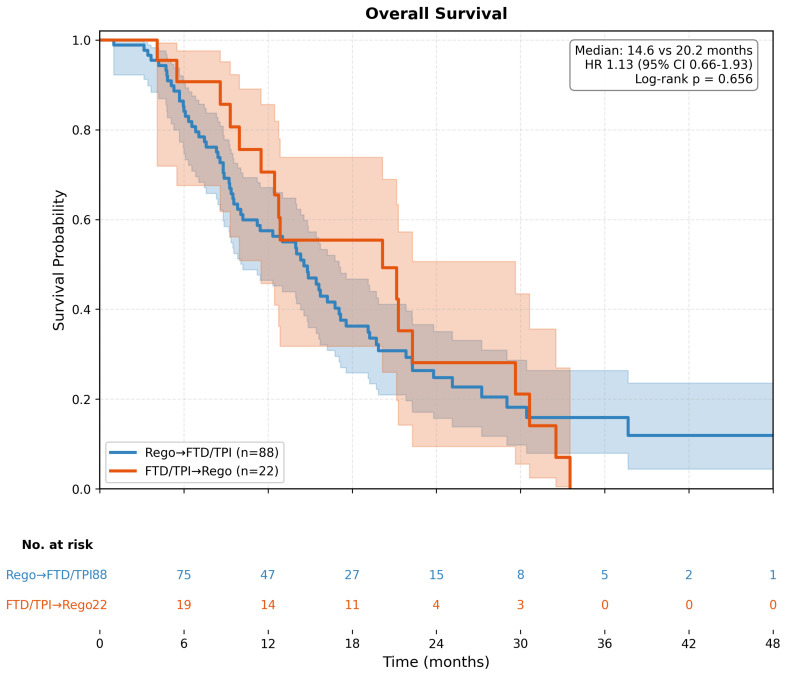
Kaplan–Meier curves for overall survival (OS) by treatment sequence (unadjusted analysis). The curves compare OS between patients who received Regorafenib → FTD/TPI (blue line, *n* = 88) versus FTD/TPI → Regorafenib (red line, *n* = 22). Shaded areas represent 95% confidence intervals. Median OS was 14.6 months (95% CI 12.5–18.2) for Rego → FTD/TPI and 20.2 months (95% CI 13.1–27.8) for FTD/TPI → Rego. The unadjusted hazard ratio was 1.13 (95% CI 0.66–1.93; log-rank *p* = 0.658). Numbers at risk are shown below the x-axis.

In the unadjusted analysis, median TTD was 6.8 months (95% CI 5.6–8.3) in the Rego → FTD/TPI group and 8.9 months (95% CI 6.4–12.5) in the FTD/TPI → Rego group. The unadjusted HR for TTD was 1.23 (95% CI 0.77–1.97; *p* = 0.389), indicating no statistically significant difference between sequences (Figure 2). Median OS was 14.6 months (95% CI 12.5–18.2) in the Rego → FTD/TPI group and 20.2 months (95% CI 13.1–27.8) in the FTD/TPI → Rego group. The unadjusted HR for OS was 1.13 (95% CI 0.66–1.93; *p* = 0.658) (Figure 3).

In the primary S-IPTW weighted analysis, the HR for TTD was 1.01 (95% CI 0.71–1.43; *p* = 0.976), and the HR for OS was 1.19 (95% CI 0.67–2.12; *p* = 0.541), neither of which reached statistical significance (Table 2, Supplementary Figure S5). Results were consistent across all analytical approaches: IPTW (TTD HR 1.01, 95% CI 0.69–1.47; OS HR 1.18, 95% CI 0.68–2.07), overlap weights (TTD HR 1.29, 95% CI 0.86–1.93; OS HR 1.13, 95% CI 0.69–1.87), and multivariable Cox regression (TTD HR 1.59, 95% CI 0.92–2.76; OS HR 1.38, 95% CI 0.75–2.55). All 95% confidence intervals crossed 1.0, indicating no statistically significant difference between treatment sequences.

### 3.4. Adverse Events

Grade 3 or higher adverse events are summarized in Table S7. Overall, any Grade 3 or higher adverse event (combining clinical and hematologic toxicities) was observed in 25 patients (22.7%), with no significant difference between the Rego → FTD/TPI and FTD/TPI → Rego groups (21.6% vs. 27.3%, *p* = 0.577). Clinical Grade 3 or higher adverse events occurred at the same rate in both groups (9.1% vs. 9.1%, *p* = 1.000). The most common clinical toxicities included hand-foot skin reaction (1.8%), thrombocytopenia (2.7%), and anemia (1.8%). Among hematologic toxicities graded from laboratory data, anemia was the most frequent (13.6% vs. 18.2%, *p* = 0.735), while neutropenia (2.3% vs. 0%, *p* = 1.000) and thrombocytopenia (4.5% vs. 0%, *p* = 0.582) were uncommon. These findings suggest that the two treatment sequences are associated with comparable severe toxicity profiles in this Asian cohort.

### 3.5. Subgroup Analyses

**Figure 4:**
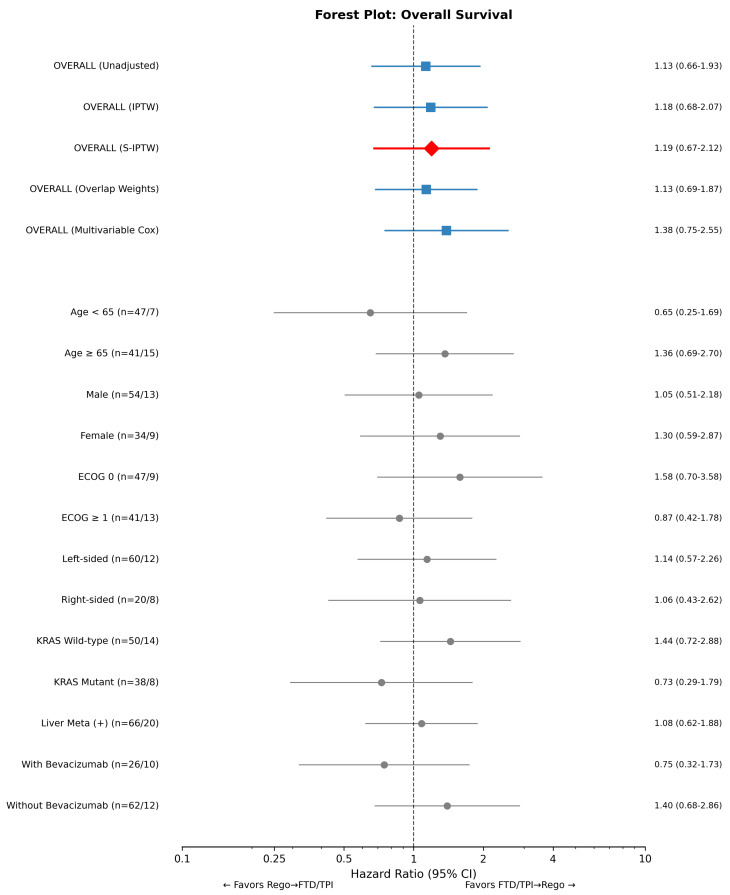
Forest plot for overall survival (OS). The plot displays hazard ratios (HRs) and 95% confidence intervals (CIs) for overall survival comparing Regorafenib → FTD/TPI versus FTD/TPI → Regorafenib (reference). Results are shown for primary analyses (unadjusted, IPTW, S-IPTW, overlap weights, and multivariable Cox regression) and exploratory subgroup analyses stratified by age, sex, ECOG performance status, primary tumor location, KRAS mutation status, presence of liver metastasis, and bevacizumab combination use. HR > 1 indicates worse outcomes for Rego → FTD/TPI sequence. The vertical dashed line represents HR = 1.0 (no difference). All 95% CIs crossed 1.0, indicating no statistically significant differences. Blue squares indicate primary analyses; the red diamond indicates the S-IPTW estimate; gray circles indicate subgroup analyses. Horizontal lines represent 95% CIs. Abbreviations: CI, confidence interval; ECOG, Eastern Cooperative Oncology Group; FTD/TPI, trifluridine/tipiracil; HR, hazard ratio; IPTW, inverse probability of treatment weighting; KRAS, Kirsten rat sarcoma viral proto-oncogene; OS, overall survival; Rego, regorafenib; S-IPTW, stabilized inverse probability of treatment weighting.

Forest plots displaying the results of primary and subgroup analyses are shown in Figure 4 and Supplementary Figure S3. The HR point estimates varied across subgroups but showed no statistically significant heterogeneity (all interaction *p*-values > 0.05; Supplementary Table S3). In the exploratory bevacizumab subgroup analysis, patients receiving FTD/TPI with bevacizumab showed a numerically lower OS HR (0.75; 95% CI 0.32–1.73) compared with those without bevacizumab (1.40; 95% CI 0.68–2.86), though neither reached statistical significance and the interaction was not significant. We additionally explored whether KRAS mutation status modified the effect of treatment sequencing (Supplementary Table S8). In the KRAS wild-type subgroup (*n* = 64), point estimates numerically favored the FTD/TPI → Rego sequence for both TTD (HR 1.52, 95% CI 0.83–2.76) and OS (HR 1.44, 95% CI 0.72–2.88), whereas in the KRAS mutant subgroup (*n* = 46), point estimates favored the Rego → FTD/TPI sequence (TTD HR 0.74, 95% CI 0.33–1.63; OS HR 0.73, 95% CI 0.29–1.79). However, the treatment-by-KRAS interaction was not statistically significant for either TTD (*p* = 0.122) or OS (*p* = 0.212), and these findings should be interpreted as hypothesis-generating only.

### 3.6. Sensitivity Analyses

To address the potential influence of treatment era on outcomes, we divided the cohort at the median treatment start date (November 2021) into Early (2017–2021, *n* = 55) and Late (2021–2025, *n* = 55) eras (Supplementary Table S9). No significant difference in TTD or OS between sequences was observed in either era (Early: TTD HR 1.01, 95% CI 0.49–2.09; OS HR 1.01, 95% CI 0.49–2.08; Late: TTD HR 1.47, 95% CI 0.78–2.75; OS HR 1.16, 95% CI 0.52–2.61), and the treatment-by-era interaction was not statistically significant for TTD (*p* = 0.413) or OS (*p* = 0.763). Furthermore, a Cox model additionally adjusting for treatment era yielded results consistent with the primary analysis (TTD HR 1.27, 95% CI 0.79–2.04; OS HR 1.08, 95% CI 0.63–1.84), confirming that treatment era does not materially confound the treatment sequence comparison. The E-value analysis indicated moderate sensitivity to unmeasured confounding (Supplementary Table S4). For the S-IPTW weighted OS analysis (HR 1.19), the E-value was 1.68 for the point estimate and 1.00 for the confidence interval.

### 3.7. Reasons for Treatment Discontinuation

The reasons for second-agent discontinuation are summarized in Table S10. Disease progression was the most common reason for discontinuation overall (74.5%), with a frequency of 80.7% in the Rego → FTD/TPI group and 50.0% in the FTD/TPI → Rego group. The FTD/TPI → Rego group had a higher proportion of discontinuation due to death (27.3% vs. 15.9%). Toxicity-related discontinuation was rare, occurring in only one patient (0.9%) in the FTD/TPI → Rego group who discontinued regorafenib due to Grade 3 hand-foot skin reaction. Regarding first-agent discontinuation, disease progression accounted for nearly all cases (99.1%), with only one patient discontinuing regorafenib due to Grade 3 hypertension.

## 4. Discussion

In this retrospective cohort study of 110 patients with mCRC who received both regorafenib and FTD/TPI sequentially, the hazard ratios for TTD (HR 1.01) and OS (HR 1.19) were close to unity, with confidence intervals spanning the null value across all analytical approaches. These findings are consistent with the primary results of the recently published SOREGATT/PRODIGE 68 trial. In our real-world cohort at Tri-Service General Hospital, we also found no statistically significant difference in clinical outcomes between the two treatment sequences of regorafenib and FTD/TPI. The adjusted hazard ratios provide important validation of these observations in an Asian real-world setting [24].

The consistency between our findings and the SOREGATT trial results is noteworthy and clinically relevant. The SOREGATT trial, a randomized phase II study conducted in France, found no significant difference in OS between the two treatment sequences despite observing higher treatment feasibility with the FTD/TPI-first approach [24]. Our real-world data from Taiwan independently corroborate these findings in a different geographic and healthcare context. Similarly, Moriwaki et al. reported comparable efficacy between regorafenib and FTD/TPI when used as primary or secondary treatments in a Japanese multicenter cohort [35]. The convergence of evidence from randomized trials and multiple real-world cohorts across different countries strengthens the conclusion that neither sequence offers a definitive survival advantage over the other.

Recently, Ahn et al. reported a retrospective analysis of sequential regorafenib and FTD/TPI treatment from US community clinical practice, similarly concluding that no significant difference in clinical outcomes was observed between the two sequencing strategies [36]. Our study extends these findings in several important ways. First, we provide real-world evidence from an Asian (Taiwanese) population, where patient characteristics, treatment access patterns, and pharmacogenomic backgrounds may differ from Western cohorts. Second, we employed propensity score-based causal inference methods, including stabilized inverse probability of treatment weighting and overlap weighting, to more rigorously adjust for treatment selection bias compared with descriptive analysis alone. Third, our KRAS biomarker interaction analysis suggests a potential differential effect of treatment sequencing by molecular subtype, providing a hypothesis-generating framework for future investigation. Together with the prospective SOREGATT trial and the US-based Ahn et al. study, our findings contribute to the growing body of evidence supporting an individualized, patient-centered approach to treatment sequencing in refractory mCRC.

The clinical implications of our findings support an individualized approach to treatment sequencing in refractory mCRC. Given the absence of a clear survival difference, clinicians can reasonably select the initial agent based on patient-specific factors including expected toxicity profiles, patient preferences, and clinical circumstances. Regorafenib is associated with notable dermatologic toxicity (hand-foot skin reaction, rash) and constitutional symptoms (fatigue, anorexia), which may be dose-limiting for some patients [13,14]. In contrast, FTD/TPI primarily causes myelosuppression, with grade 3–4 neutropenia occurring in approximately 38% of patients in the RECOURSE trial, though febrile neutropenia was relatively uncommon (4%) [16]. Patients with pre-existing dermatologic conditions or concerns about skin toxicity may be better suited to initiate with FTD/TPI, while those with baseline cytopenias or concerns about bone marrow suppression may benefit from starting with regorafenib.

It is important to contextualize our findings within the current treatment landscape, which has been transformed by the SUNLIGHT trial demonstrating that combining FTD/TPI with bevacizumab significantly improves OS compared with FTD/TPI alone (median 10.8 vs. 7.5 months; HR 0.61, 95% CI 0.49–0.77) [22,23]. This combination has now been adopted as a preferred third-line option in updated clinical guidelines. In our exploratory subgroup analysis, patients receiving FTD/TPI with bevacizumab showed a numerically lower hazard ratio for OS compared with those receiving FTD/TPI monotherapy, an observation consistent with the SUNLIGHT trial findings. However, the small sample sizes preclude definitive conclusions. As the FTD/TPI plus bevacizumab combination becomes increasingly adopted in clinical practice, future studies examining optimal treatment sequencing should incorporate this regimen as a distinct treatment option. Whether the addition of bevacizumab modifies the relative efficacy of different sequencing strategies remains an important unanswered question that warrants dedicated investigation. For patients who are candidates for FTD/TPI plus bevacizumab, sequencing decisions may therefore need to consider factors beyond those applicable to monotherapy.

The marked imbalance in treatment sequence distribution in our cohort (80% vs. 20%) likely reflects the chronological pattern of drug availability and reimbursement in Taiwan, where regorafenib was approved before FTD/TPI. This introduces potential era effects, as patients in the FTD/TPI-first group were likely treated in more recent years with potential improvements in supportive care and subsequent treatment options. We addressed this through comprehensive propensity score adjustment including bevacizumab combination status, which achieved good covariate balance (11 of 14 covariates with |SMD| < 0.1). Nevertheless, residual confounding from unmeasured factors cannot be excluded.

From a mechanistic perspective, the similar efficacy of both sequences may be explained by the non-overlapping mechanisms of action between the two agents. FTD/TPI acts through direct incorporation into DNA causing strand breaks, while regorafenib exerts its effects primarily through inhibition of angiogenic and oncogenic kinases [12,15]. The mechanistic independence suggests that sequential use of either agent after progression on the other remains clinically valid, regardless of order. Preclinical studies have shown that regorafenib and FTD/TPI may have synergistic effects in certain contexts, suggesting that exposure to both agents, rather than their sequence, may be the more important factor [37].

Our adverse event analysis revealed comparable Grade 3 or higher toxicity rates between the two treatment sequences (21.6% vs. 27.3%, *p* = 0.577), suggesting that neither sequence is associated with a clearly more favorable safety profile. Anemia was the most common hematologic toxicity in both groups, consistent with the known myelosuppressive effects of FTD/TPI. The similar overall toxicity rates between sequences support the notion that treatment selection can be guided by individual patient factors rather than anticipated toxicity differences between the two sequencing strategies. However, we note that the hematologic toxicity rates, particularly neutropenia (1.8%), were lower than reported in pivotal clinical trials such as RECOURSE (~38% Grade 3+ neutropenia). This is likely attributable to the retrospective nature of our study and reliance on available laboratory data rather than systematic nadir CBC monitoring during each treatment cycle, potentially underestimating true hematologic toxicity rates.

An exploratory biomarker analysis revealed a qualitative interaction between KRAS mutation status and treatment sequencing benefit, although this did not reach statistical significance. In the KRAS wild-type subgroup, point estimates consistently favored the FTD/TPI-first sequence, whereas in the KRAS mutant subgroup, the direction reversed to favor the regorafenib-first sequence. While these results must be interpreted with caution given the small subgroup sizes and non-significant interaction tests, this pattern is biologically plausible. Regorafenib, as a multikinase inhibitor targeting the RAS/RAF/MEK pathway, may exert differential effects depending on the KRAS-driven signaling context. If validated in larger, adequately powered studies, KRAS mutation status could serve as a predictive biomarker to guide personalized treatment sequencing decisions in refractory mCRC.

Our treatment era sensitivity analysis demonstrated that the non-significant difference between sequences was consistent across both early (2017–2021) and late (2021–2025) eras, with no significant Treatment × Era interaction (TTD *p* = 0.413; OS *p* = 0.763). Furthermore, a Cox model adjusting for treatment era produced results consistent with the primary analysis. This finding mitigates the concern that evolving treatment strategies or supportive care practices over the study period may have differentially influenced outcomes between the two sequences.

The distribution of discontinuation reasons showed notable descriptive differences between sequences. The FTD/TPI → Rego group had a lower proportion of disease progression as the reason for second-agent discontinuation (50.0% vs. 80.7%) and a correspondingly higher proportion of death-related discontinuation (27.3% vs. 15.9%). This pattern may reflect the generally poorer prognosis of patients reaching the second salvage-line agent in the FTD/TPI-first sequence, or alternatively may be influenced by the natural history of more aggressive disease in this subgroup. Importantly, toxicity was rarely the proximate cause of treatment discontinuation in either sequence (0.9%), reinforcing that both sequencing strategies are clinically feasible from a tolerability standpoint.

Our study has several strengths. First, we employed multiple propensity score-based methods (IPTW, S-IPTW, overlap weights) and multivariable regression to address confounding bias, with all approaches yielding consistent results supporting the robustness of our findings. Second, the inclusion of bevacizumab combination as a covariate reflects contemporary treatment patterns following the SUNLIGHT trial. Third, the use of overlap weights is particularly appropriate for our sample given the imbalanced group sizes and potential positivity violations with extreme propensity scores [31,32]. Fourth, our study population represents real-world clinical practice in Taiwan, providing important validation that findings from Western trials generalize to Asian populations.

Several important limitations warrant consideration. First, the modest sample size, particularly in the FTD/TPI-first group (*n* = 22), limits statistical power to detect potentially clinically meaningful differences between sequences, raising the possibility of a Type II error. The 4:1 group imbalance reflects real-world prescribing patterns in Taiwan, where regorafenib was more commonly used as initial salvage therapy during the study period. The wide confidence intervals observed across analyses (TTD HR 1.01, 95% CI 0.71–1.43; OS HR 1.19, 95% CI 0.67–2.12) further underscore this limitation, and clinically relevant differences between sequences cannot be definitively excluded. Larger, prospective, multi-center studies are warranted to validate these findings with adequate statistical power. Second, the retrospective observational design is inherently subject to selection bias and unmeasured confounding. Despite propensity score adjustment, residual confounding from unmeasured variables—such as performance status trajectory, nutritional status, and patient preference—or imperfectly balanced measured covariates, including age and liver metastasis prevalence, cannot be excluded. The use of overlap weighting, which naturally downweights individuals with extreme propensity scores and focuses inference on the population of clinical equipoise, mitigates but does not eliminate this concern. The E-value of 1.68 for the OS point estimate indicates that an unmeasured confounder would need only modest association strength to explain away the observed effect. Nevertheless, our multivariable Cox regression sensitivity analysis yielded results consistent with the primary analysis, providing additional reassurance regarding the robustness of our findings. Third, the imbalanced group distribution likely reflects chronological bias from drug availability timing, introducing potential era effects that we could not fully address. Fourth, we did not have complete data on treatment response (RECIST criteria) or quality of life outcomes. Adverse event data were collected retrospectively through chart review and available laboratory records, which may underestimate the true incidence of treatment-related toxicities compared with prospective monitoring. In particular, hematologic toxicity rates—especially neutropenia—were lower than expected based on pivotal trial data, likely reflecting the absence of systematic nadir CBC monitoring in routine clinical practice. These limitations in toxicity ascertainment may have attenuated our ability to detect meaningful differences in adverse event profiles between sequences. Fifth, our study was conducted at a single tertiary medical center in Taiwan, which may limit generalizability to other practice settings. External validation through multi-center studies involving diverse Asian populations is needed. Nevertheless, the consistency of our findings with those of the prospective SOREGATT trial and the US-based community practice study by Ahn et al. provides indirect cross-cultural validation, suggesting that the absence of a significant difference in sequencing outcomes may represent a robust finding across different populations and healthcare settings.

## 5. Conclusions

In this retrospective cohort of 110 patients with refractory mCRC, no statistically significant difference in TTD or OS was observed between the Rego → FTD/TPI and FTD/TPI → Rego sequences after propensity score adjustment. However, the wide confidence intervals preclude definitive conclusions, and clinically meaningful differences cannot be excluded. Adverse event profiles were comparable between the two sequences. Exploratory analysis revealed a qualitative interaction between KRAS mutation status and sequencing benefit, suggesting potential utility as a predictive biomarker. Treatment era did not significantly confound the primary findings. These results support an individualized approach to treatment sequencing in refractory mCRC, guided by patient-specific factors including comorbidities, toxicity profiles, and preferences. Larger, prospective, multi-center studies—ideally incorporating molecular biomarker stratification—are warranted to definitively establish optimal sequencing strategies and identify patients who may benefit from a specific treatment order.

## Figures and Tables

**Table 1:** Baseline Characteristics of Patients by Treatment Sequence.

Category	Variable	Overall (*n* = 110)	Rego → FTD/TPI (*n* = 88)	FTD/TPI → Rego (*n* = 22)	SMD
Demographics	Age, years, mean ± SD	63.9 ± 10.2	63.3 ± 10.3	66.2 ± 9.8	−0.28
	Male, *n* (%)	67 (60.9)	54 (61.4)	13 (59.1)	0.03
	ECOG PS 0, *n* (%)	56 (50.9)	47 (53.4)	9 (40.9)	
	ECOG PS 1, *n* (%)	41 (37.3)	31 (35.2)	10 (45.5)	
	ECOG PS ≥ 2, *n* (%)	13 (11.8)	10 (11.4)	3 (13.6)	
	CCI, mean ± SD	7.8 ± 2.0	7.8 ± 1.8	8.0 ± 2.5	−0.08
Tumor Characteristics	Left-sided, *n* (%)	72 (65.5)	60 (68.2)	12 (54.5)	
	Right-sided, *n* (%)	28 (25.5)	20 (22.7)	8 (36.4)	−0.21
	Rectum, *n* (%)	10 (9.1)	8 (9.1)	2 (9.1)	0.00
	KRAS mutation, *n* (%)	46 (41.8)	38 (43.2)	8 (36.4)	0.10
Metastatic Sites	Liver, *n* (%)	86 (78.2)	66 (75.0)	20 (90.9)	−0.30
	Lung, *n* (%)	75 (68.2)	60 (68.2)	15 (68.2)	0.00
	Bone, *n* (%)	11 (10.0)	9 (10.2)	2 (9.1)	0.03
Treatment History	Prior lines, mean ± SD	3.0 ± 0.8	3.0 ± 0.8	2.8 ± 0.6	0.26
	Prior anti-EGFR, *n* (%)	58 (52.7)	46 (52.3)	12 (54.5)	−0.03
	Prior colectomy, *n* (%)	65 (59.1)	51 (58.0)	14 (63.6)	−0.08
Study Treatment	Regorafenib dose, mg, mean ± SD	94.9 ± 35.1	97.7 ± 36.3	83.6 ± 27.4	0.44
	FTD/TPI dose, mg, mean ± SD	76.4 ± 33.5	76.0 ± 34.8	77.7 ± 28.8	−0.05
	FTD/TPI + Bevacizumab, *n* (%)	36 (32.7)	26 (29.5)	10 (45.5)	−0.23

Abbreviations: CCI, Charlson Comorbidity Index; ECOG PS, Eastern Cooperative Oncology Group performance status; FTD/TPI, trifluridine/tipiracil; Rego, regorafenib; SMD, standardized mean difference. Note: |SMD| < 0.1 indicates adequate balance between groups.

**Table 2:** Primary and Sensitivity Analysis Results.

Outcome	Method	HR (95% CI)	*p*-Value
Time to Discontinuation	Unadjusted	1.23 (0.77–1.97)	0.389
	IPTW	1.01 (0.69–1.47)	0.959
	S-IPTW (Primary)	1.01 (0.71–1.43)	0.976
	Overlap Weights	1.29 (0.86–1.93)	0.217
	Multivariable Cox	1.59 (0.92–2.76)	0.100
Overall Survival	Unadjusted	1.13 (0.66–1.93)	0.658
	IPTW	1.18 (0.68–2.07)	0.553
	S-IPTW (Primary)	1.19 (0.67–2.12)	0.541
	Overlap Weights	1.13 (0.69–1.87)	0.625
	Multivariable Cox	1.38 (0.75–2.55)	0.296

Abbreviations: CI, confidence interval; HR, hazard ratio; IPTW, inverse probability of treatment weighting; S-IPTW, stabilized IPTW. Note: HR > 1 indicates worse outcome for Rego → FTD/TPI sequence. Reference group: FTD/TPI → Rego.

## Data Availability

The data presented in this study are available upon request from the corresponding author due to privacy and ethical restrictions.

## Supplementary Materials

The following supporting information can be downloaded at: https://www.mdpi.com/article/10.3390/life16040564/s1, Figure S1: Propensity score distribution by treatment sequence; Figure S2: Covariate balance before and after S-IPTW weighting (Love plot); Figure S3: Forest plot for time to discontinuation; Figure S4: Distribution of propensity score weights; Figure S5: S-IPTW weighted Kaplan–Meier curves; Table S1: Propensity score model for treatment sequence assignment; Table S2: Covariate balance before and after S-IPTW weighting; Table S3: Subgroup analysis results; Table S4: E-value sensitivity analysis for unmeasured confounding; Table S5: Distribution of propensity score weights; Table S6: Multivariable Cox regression full model results; Table S7: Grade 3 or Higher Adverse Events by Treatment Sequence; Table S8: KRAS Subgroup Analysis with Biomarker-Treatment Interaction; Table S9: Treatment Era Sensitivity Analysis; Table S10: Reasons for Second-Agent Discontinuation by Treatment Sequence.

## Abbreviations

The following abbreviations are used in this manuscript:

CCI	Charlson Comorbidity Index
CRC	Colorectal Cancer
ECOG PS	Eastern Cooperative Oncology Group Performance Status
FTD/TPI	Trifluridine/tipiracil
IPTW	Inverse Probability of Treatment Weighting
mCRC	Metastatic Colorectal Cancer
S-IPTW	Stabilized Inverse Probability of Treatment Weighting
TTD	Time to Treatment Discontinuation
VEGF	Vascular Endothelial Growth Factor
VEGFR	Vascular Endothelial Growth Factor Receptor
