# Supplementary material for: Sequential Treatment with Regorafenib and Trifluridine/Tipiracil in Refractory Metastatic Colorectal Cancer
Source: Life (Basel). 2026 Mar 30;16(4):564. doi: 10.3390/life16040564 (PMC13117735; doi:10.3390/life16040564)
Supplement: Supplementary file 1 [file life-16-00564-s001.zip › life-4199793-Supplementary Material.pdf]

Supplementary Material

Sequential treatment with regorafenib and trifluridine/tipiracil in refractory metastatic colorectal cancer

Min-Chi Cheng, Po-Huang Chen, Yu-Guang Chen, Shiue-Wei Lai, Jia-Hong Chen, Ming-Shen Dai, Ping-Ying Chang

Life

Table of Contents

Tables

Table S1: Propensity Score Model for Treatment Sequence Assignment
Table S2: Covariate Balance Before and After S-IPTW Weighting
Table S3: Subgroup Analysis Results
Table S4: E-value Sensitivity Analysis for Unmeasured Confounding
Table S5: Distribution of Propensity Score Weights
Table S6: Multivariable Cox Regression – Full Model Results
Table S7: Grade 3 or Higher Adverse Events by Treatment Sequence
Table S8: KRAS Subgroup Analysis with Biomarker-Treatment Interaction
Table S9: Treatment Era Sensitivity Analysis
Table S10: Reasons for Second-Agent Discontinuation by Treatment Sequence

Figures

Figure S1: Propensity Score Distribution
Figure S2: Love Plot – Covariate Balance
Figure S3: Forest Plot – Time to Discontinuation
Figure S4: Propensity Score Weight Distribution
Figure S5: Weighted Kaplan-Meier Curves

Table S1. Propensity Score Model for Treatment Sequence Assignment

Covariate	Coefficient	SE	OR (95% CI)	p-value
Intercept	1.624	0.29	–	<0.001
Age	-0.209	0.301	0.81 (0.45–1.46)	0.487
Sex_male	0.155	0.285	1.17 (0.67–2.04)	0.586
ECOG	-0.424	0.271	0.65 (0.38–1.11)	0.118
CCI	-0.088	0.286	0.92 (0.52–1.60)	0.758
Location_Right	-0.238	0.261	0.79 (0.47–1.32)	0.363
Location_Rectum	-0.036	0.287	0.96 (0.55–1.69)	0.900
KRAS_mut	0.216	0.42	1.24 (0.54–2.83)	0.608
Meta_Lung	-0.01	0.281	0.99 (0.57–1.72)	0.973
Meta_Liver	-0.461	0.368	0.63 (0.31–1.30)	0.211
Meta_Bone	0.201	0.29	1.22 (0.69–2.16)	0.488
Prior_lines	0.309	0.275	1.36 (0.79–2.33)	0.261
Prior_EGFR	0.064	0.411	1.07 (0.48–2.39)	0.877
Colectomy	-0.002	0.284	1.00 (0.57–1.74)	0.994
Lonsurf_with_beva	-0.538	0.289	0.58 (0.33–1.03)	0.063

Note: *Outcome = Probability of receiving Rego→FTD/TPI sequence. Model includes FTD/TPI + Bevacizumab as covariate. Coefficients are standardized.*

Table S2. Covariate Balance Before and After S-IPTW Weighting

Covariate	Unadjusted SMD	S-IPTW SMD
Age	-0.28	0.24
Sex_male	0.03	0.00
ECOG	-0.26	-0.08
CCI	-0.08	0.21
Location_Right	-0.21	-0.02
Location_Rectum	0.00	0.06
KRAS_mut	0.10	-0.21
Meta_Lung	0.00	0.01
Meta_Liver	-0.30	0.06
Meta_Bone	0.03	0.10
Prior_lines	0.26	-0.06
Prior_EGFR	-0.03	0.07
Colectomy	-0.08	-0.02
Lonsurf_with_beva	-0.23	-0.05

Note: *SMD, standardized mean difference; S-IPTW, stabilized inverse probability of treatment weighting. |SMD| < 0.1 indicates adequate balance. 11/14 covariates achieved balance after S-IPTW weighting.*

Table S3. Subgroup Analysis Results

Subgroup	N (Rego/FTD)	TTD HR (95% CI)	OS HR (95% CI)	Interaction p
Age < 65	47/7	0.74 (0.33–1.68)	0.65 (0.25–1.69)	
Age ≥ 65	41/15	1.58 (0.87–2.88)	1.36 (0.69–2.70)	
Male	54/13	1.39 (0.76–2.56)	1.05 (0.51–2.18)	
Female	34/9	0.97 (0.45–2.07)	1.30 (0.59–2.87)	
ECOG 0	47/9	1.08 (0.52–2.25)	1.58 (0.70–3.58)	
ECOG ≥ 1	41/13	1.47 (0.78–2.78)	0.87 (0.42–1.78)	
Left-sided	60/12	1.07 (0.57–1.99)	1.14 (0.57–2.26)	
Right-sided	20/8	1.90 (0.82–4.42)	1.06 (0.43–2.62)	
KRAS Wild-type	50/14	1.52 (0.83–2.76)	1.44 (0.72–2.88)	
KRAS Mutant	38/8	0.74 (0.33–1.63)	0.73 (0.29–1.79)	
Liver Meta (+)	66/20	1.23 (0.74–2.03)	1.08 (0.62–1.88)	
With Bevacizumab	26/10	1.02 (0.48–2.14)	0.75 (0.32–1.73)	
Without Bevacizumab	62/12	1.37 (0.73–2.56)	1.40 (0.68–2.86)	

Note: *CI, confidence interval; HR, hazard ratio; OS, overall survival; TTD, time to discontinuation. All interaction p-values > 0.05.*

Table S4. E-value Sensitivity Analysis for Unmeasured Confounding

Outcome	Method	HR	E-value (point)	E-value (CI)
TTD	S-IPTW	1.01	1.08	1.00
OS	S-IPTW	1.19	1.68	1.00

Note: *CI, confidence interval; HR, hazard ratio; S-IPTW, stabilized inverse probability of treatment weighting. E-value represents the minimum strength of association an unmeasured confounder would need with both treatment and outcome to explain away the observed effect.*

Table S5. Distribution of Propensity Score Weights

Method	Mean	SD	Min	P1	Median	P99	Max
IPTW (truncated)	2.00	2.37	1.03	1.03	1.27	15.16	15.38
S-IPTW (truncated)	1.00	0.39	0.37	0.37	0.92	3.03	3.08
Overlap Weights	0.28	0.25	0.02	0.03	0.21	0.94	0.97

Note: *IPTW, inverse probability of treatment weighting; S-IPTW, stabilized IPTW; P1, 1st percentile; P99, 99th percentile; SD, standard deviation.*

Table S6. Multivariable Cox Regression – Full Model Results

Variable	TTD HR (95% CI)	TTD p	OS HR (95% CI)	OS p
Treatment (Rego→FTD/TPI)	1.59 (0.92–2.76)	0.100	1.38 (0.75–2.55)	0.296
Age	0.97 (0.95–0.99)	0.014	0.99 (0.96–1.01)	0.336
Sex_male	0.68 (0.43–1.08)	0.104	0.67 (0.40–1.12)	0.130
ECOG	1.12 (0.87–1.46)	0.381	1.15 (0.85–1.55)	0.357
CCI	1.21 (1.08–1.35)	0.001	1.17 (1.02–1.33)	0.021
Location_Right	2.42 (1.44–4.04)	<0.001	1.29 (0.74–2.25)	0.362
Location_Rectum	2.69 (1.26–5.76)	0.011	0.87 (0.26–2.93)	0.828
KRAS_mut	1.45 (0.74–2.86)	0.282	2.65 (1.16–6.05)	0.021
Meta_Lung	0.89 (0.56–1.42)	0.630	0.54 (0.32–0.91)	0.021
Meta_Liver	0.60 (0.34–1.06)	0.077	1.66 (0.81–3.39)	0.165
Meta_Bone	2.20 (1.06–4.53)	0.033	1.76 (0.80–3.86)	0.157
Prior_lines	0.92 (0.71–1.19)	0.512	1.11 (0.83–1.49)	0.474
Prior_EGFR	2.65 (1.37–5.15)	0.004	3.28 (1.45–7.41)	0.004
Colectomy	1.09 (0.70–1.69)	0.716	1.09 (0.66–1.78)	0.737
Lonsurf_with_beva	0.95 (0.60–1.50)	0.826	0.85 (0.49–1.48)	0.562

Note: *CI, confidence interval; HR, hazard ratio; OS, overall survival; TTD, time to discontinuation.*

Table S7. Grade 3 or Higher Adverse Events by Treatment Sequence

Adverse Event	Rego→FTD/TPI (n=88)	FTD/TPI→Rego (n=22)	Total (N=110)	p value
**Any clinical Grade ≥3 AE**	**8 (9.1%)**	**2 (9.1%)**	**10 (9.1%)**	**1.000**
HFSR	1 (1.1%)	1 (4.5%)	2 (1.8%)	–
Anemia (clinical)	2 (2.3%)	0 (0%)	2 (1.8%)	–
Thrombocytopenia (clinical)	3 (3.4%)	0 (0%)	3 (2.7%)	–
Mucositis	0 (0%)	1 (4.5%)	1 (0.9%)	–
Fatigue	1 (1.1%)	0 (0%)	1 (0.9%)	–
Weight loss	1 (1.1%)	0 (0%)	1 (0.9%)	–
**Hematologic Grade ≥3 (lab-based)**				
Neutropenia	2 (2.3%)	0 (0%)	2 (1.8%)	1.000
Anemia	12 (13.6%)	4 (18.2%)	16 (14.5%)	0.735
Thrombocytopenia	4 (4.5%)	0 (0%)	4 (3.6%)	0.582
Any hematologic	15 (17.0%)	4 (18.2%)	19 (17.3%)	1.000
**Any Grade ≥3 AE (combined)**	**19 (21.6%)**	**6 (27.3%)**	**25 (22.7%)**	**0.577**

Note: *AE, adverse event; HFSR, hand-foot skin reaction; CTCAE, Common Terminology Criteria for Adverse Events. Clinical adverse events were ascertained via retrospective chart review; hematologic toxicities were graded from complete blood count laboratory data per CTCAE version 5.0. p values by Fisher's exact test.*

Table S8. KRAS Subgroup Analysis with Biomarker-Treatment Interaction

Subgroup	Rego→FTD/TPI (n)	FTD/TPI→Rego (n)	TTD HR (95% CI)	TTD p	OS HR (95% CI)	OS p
KRAS wild-type	50	14	1.52 (0.83–2.76)	0.171	1.44 (0.72–2.88)	0.302
KRAS mutant	38	8	0.74 (0.33–1.63)	0.451	0.73 (0.29–1.79)	0.485
**Interaction p-value**	**–**	**–**	**–**	**0.122**	**–**	**0.212**

Note: *CI, confidence interval; HR, hazard ratio; OS, overall survival; TTD, time to discontinuation. HR > 1 indicates higher hazard in the Rego→FTD/TPI group (i.e., favoring FTD/TPI→Rego sequence). Interaction p-value from Cox proportional hazards model with multiplicative Treatment × KRAS mutation status interaction term.*

Table S9. Treatment Era Sensitivity Analysis

Analysis	Rego→FTD/TPI (n)	FTD/TPI→Rego (n)	TTD HR (95% CI)	TTD p	OS HR (95% CI)	OS p
Early era (2017–2021)	46	9	1.01 (0.49–2.09)	0.975	1.01 (0.49–2.08)	0.977
Late era (2021–2025)	42	13	1.47 (0.78–2.75)	0.234	1.16 (0.52–2.61)	0.711
**Treatment × Era interaction p**	**–**	**–**	**–**	**0.413**	**–**	**0.763**
**Era-adjusted model**	**88**	**22**	**1.27 (0.79–2.04)**	**0.329**	**1.08 (0.63–1.84)**	**0.792**

Note: *CI, confidence interval; HR, hazard ratio; OS, overall survival; TTD, time to discontinuation. The cohort was divided at the median treatment start date (November 2021). Interaction p-value from Cox model with Treatment × Era interaction term. Era-adjusted model: Cox proportional hazards regression with treatment sequence and treatment era as covariates.*

Table S10. Reasons for Second-Agent Discontinuation by Treatment Sequence

Reason for Discontinuation	Rego→FTD/TPI (n=88)	FTD/TPI→Rego (n=22)	Total (N=110)
Disease progression	71 (80.7%)	11 (50.0%)	82 (74.5%)
Death	14 (15.9%)	6 (27.3%)	20 (18.2%)
Toxicity	0 (0%)	1 (4.5%)	1 (0.9%)
Other	1 (1.1%)	0 (0%)	1 (0.9%)
Not documented	2 (2.3%)	4 (18.2%)	6 (5.5%)

Note: *Values are n (%). Disease progression was the most common reason for discontinuation in both groups. Toxicity-related discontinuation was rare, occurring in only one patient (0.9%) in the FTD/TPI→Rego group who discontinued regorafenib due to Grade 3 hand-foot skin reaction.*

Figure S1. Propensity Score Distribution by Treatment Sequence

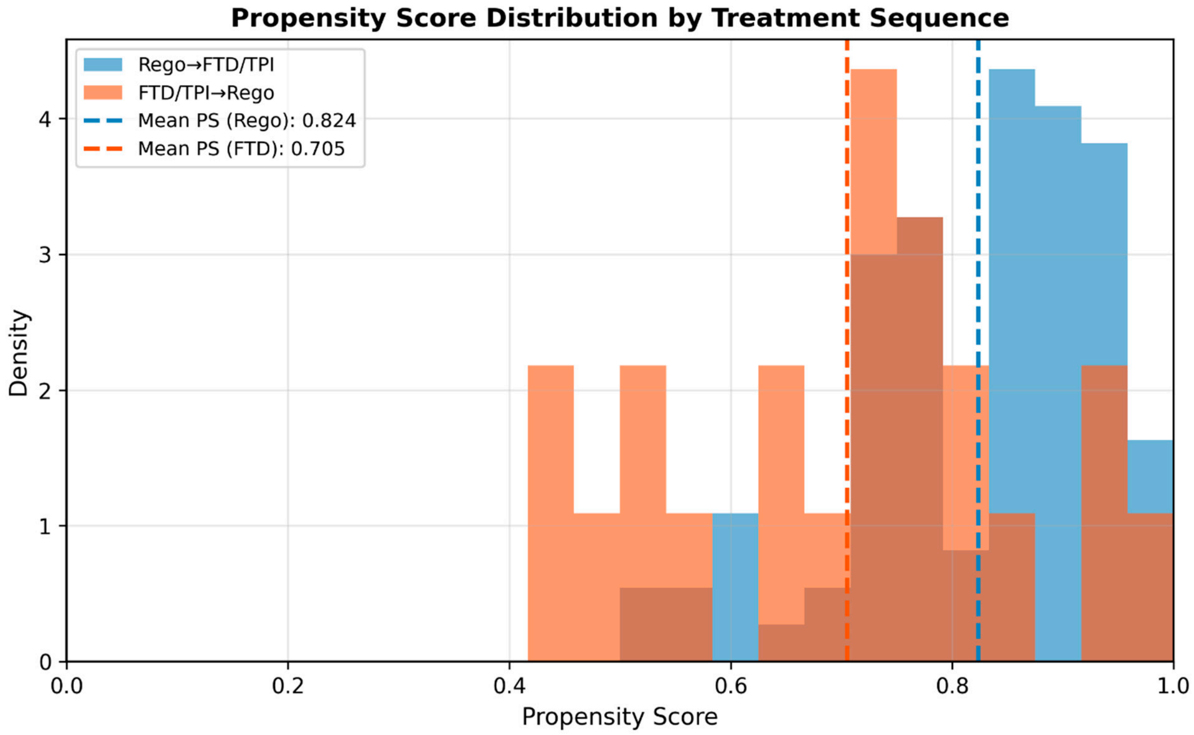

Figure S1. Propensity Score Distribution by Treatment Sequence. Histogram showing the distribution of estimated propensity scores for the Rego→FTD/TPI (blue) and FTD/TPI→Rego (orange) groups. Dashed lines indicate group means (Rego→FTD/TPI: 0.824; FTD/TPI→Rego: 0.705). Overlap between distributions supports the positivity assumption for propensity score-based analyses.

Figure S2. Love Plot – Covariate Balance Before and After S-IPTW Weighting

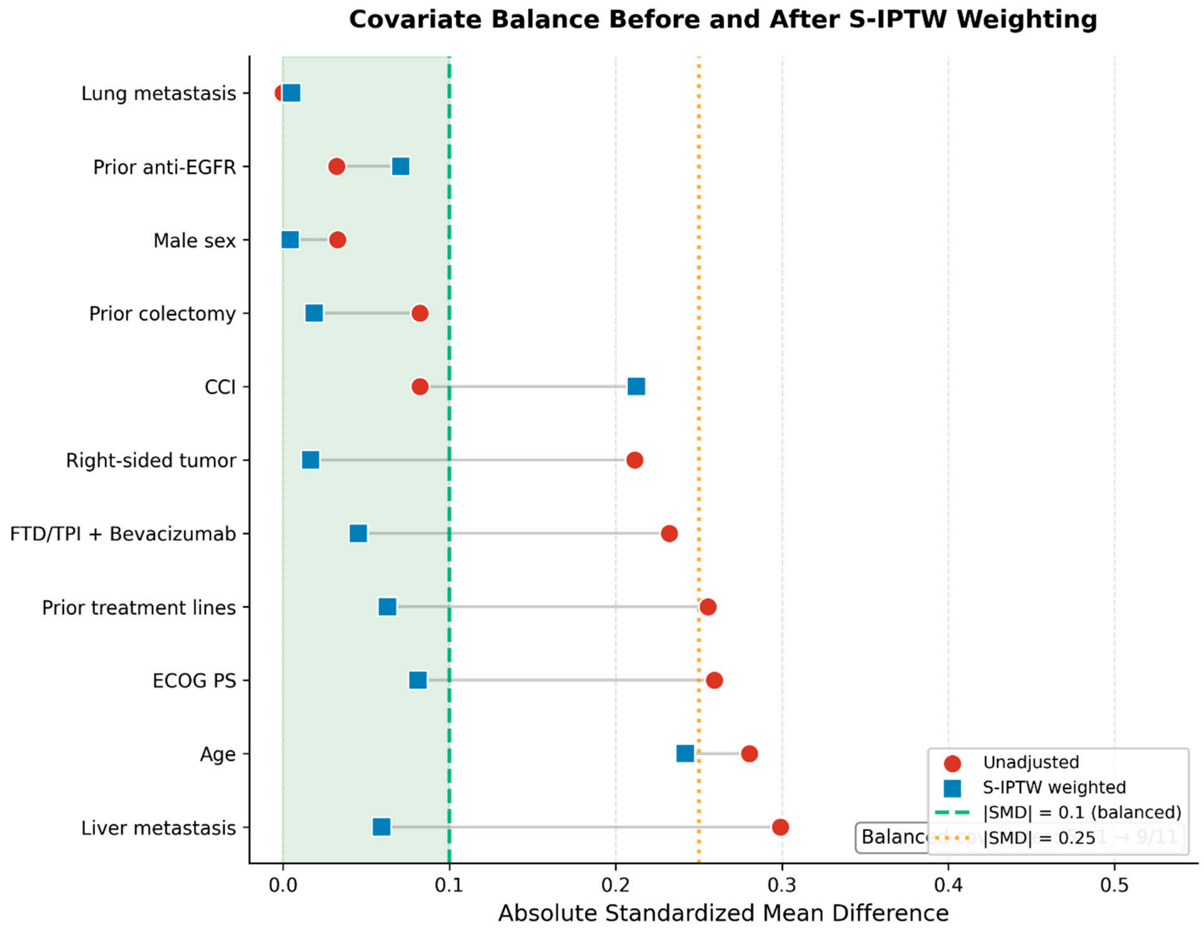

Figure S2. Love Plot – Covariate Balance Before and After S-IPTW Weighting. Love plot showing absolute standardized mean differences (SMD) for all covariates before (red circles) and after (blue squares) stabilized inverse probability of treatment weighting. The green shaded area indicates |SMD| < 0.1 (adequate balance). The dashed green line marks the 0.1 threshold; the dotted orange line marks 0.25.

Figure S3. Forest Plot – Time to Discontinuation

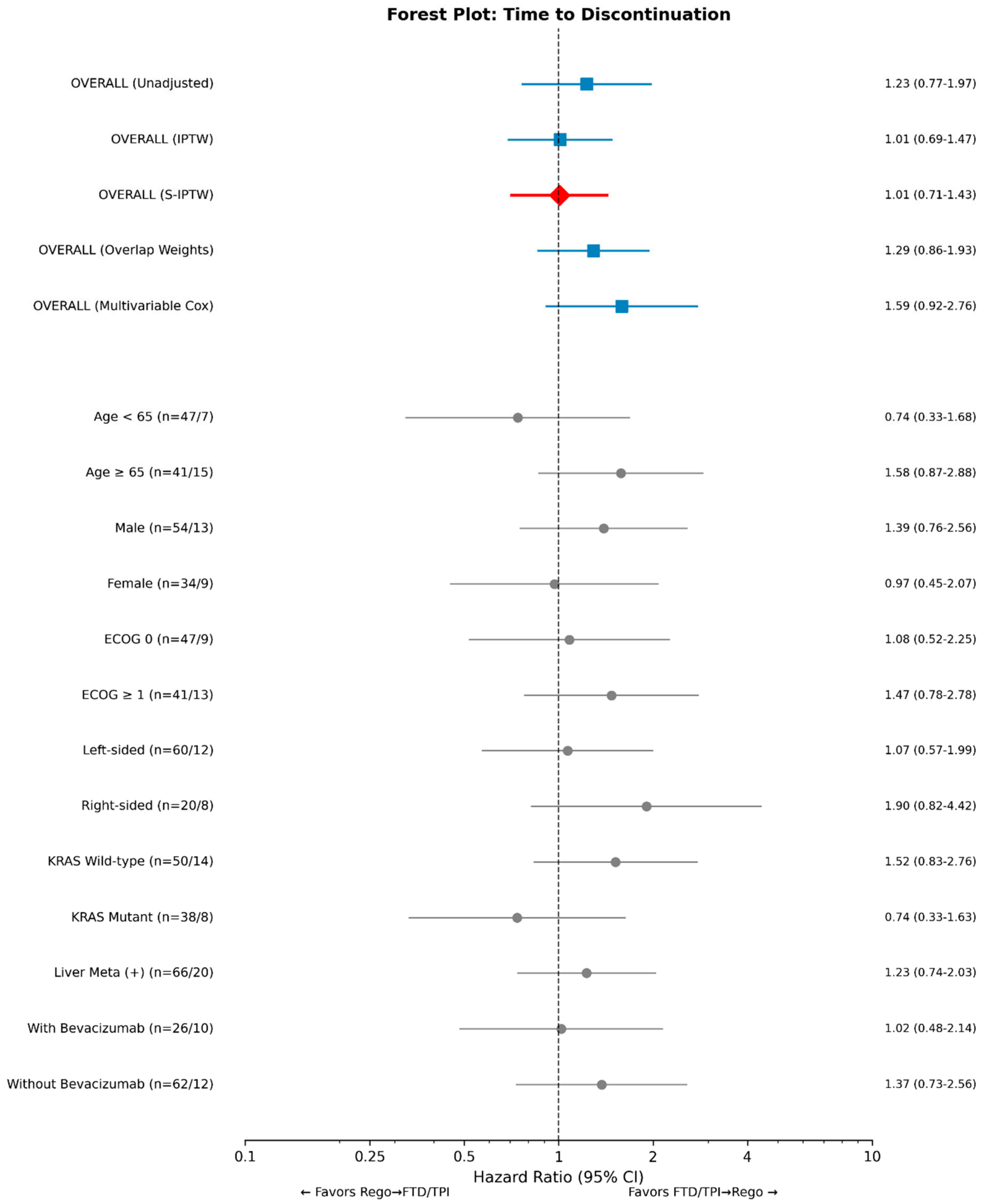

Figure S3. Forest Plot – Time to Discontinuation. Forest plot showing hazard ratios (95% CI) for time to discontinuation across multiple analytical methods (unadjusted, IPTW, S-IPTW, overlap weights, multivariable Cox) and clinical subgroups. HR > 1 favors the FTD/TPI→Rego sequence. The S-IPTW estimate (red diamond) represents the primary analysis.

Figure S4. Propensity Score Weight Distribution

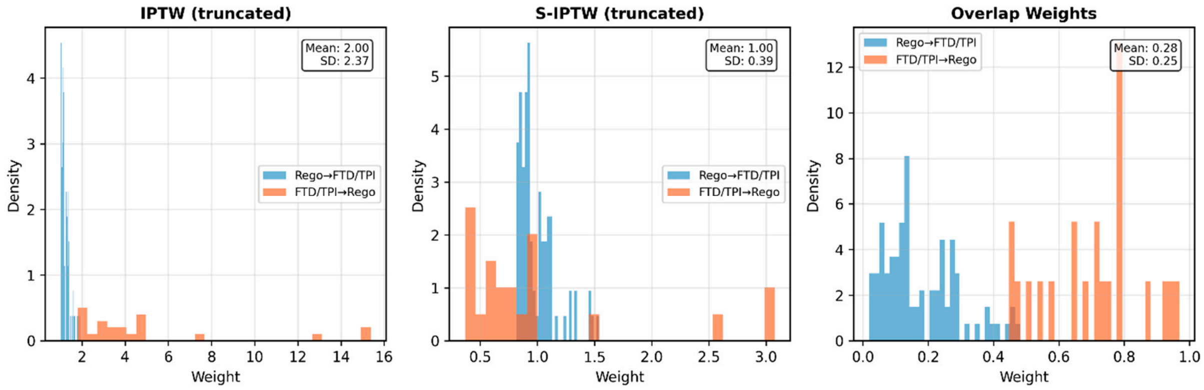

Figure S4. Propensity Score Weight Distribution. Distribution of propensity score weights for three weighting methods: IPTW (truncated at 1st/99th percentiles), S-IPTW (truncated), and overlap weights, stratified by treatment sequence. Overlap weights show the most concentrated distribution with minimal extreme values.

Figure S5. Weighted Kaplan-Meier Curves for TTD and OS

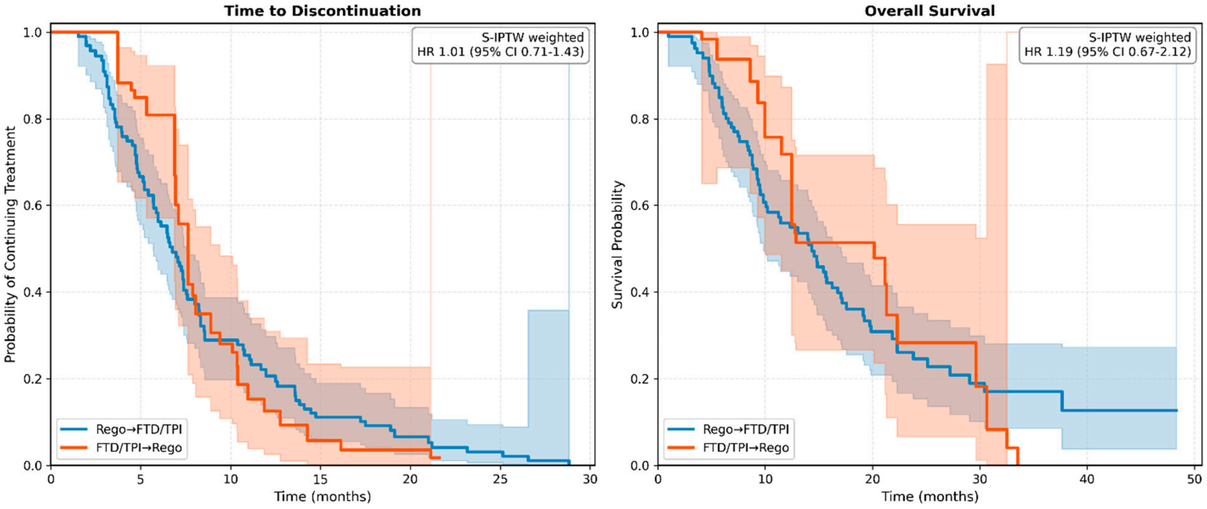

Figure S5. Weighted Kaplan-Meier Curves for TTD and OS. S-IPTW weighted Kaplan-Meier curves comparing Rego→FTD/TPI (blue) and FTD/TPI→Rego (orange) sequences for time to discontinuation (left) and overall survival (right). Shaded areas represent 95% confidence intervals. No significant difference was observed between sequences.
